# Supplementary material for: A New High-Throughput Tool to Screen Mosquito-Borne Viruses in Zika Virus Endemic/Epidemic Areas
Source: Viruses. 2019 Sep 27;11(10):904. doi: 10.3390/v11100904 (PMC6832350; doi:10.3390/v11100904)
Supplement: Supplementary file 1 [file viruses-11-00904-s001.pdf]

Table S1. List of mosquito-borne viruses, targets, primers/probe sets, and positive controls.

Family	Genus	Species	Primers/probe	Sequence (5'-3')	Target	Length (bp)	Positive controls
Flaviviridae	Flavivirus	Banzi	Banzi_F	TCT GTG CCA AAC CAG CTT AG	NS3	71	Cell culture strain SA H 336
			Banzi_R	CTC GGA TCT CAG GAG GAT TG			
			Banzi_P	TGA GAA GCT GGT TCA GTC CAT TGA CAC ACT			
		Bussuquara	BussuqV_F	GAA GGT CTT GGA GAT GGT GG	NS5	72	Cell culture strain BeAn 4073
			BussuqV_R	GTCTGTACGGGCACAACACT			
			BussuqV_P	AGC CGT GGC TTA AGA ACA AAC CTG AAT TCT			
		Dengue	Deng_1_F	AAC CCA TGG AAG CTG TAC GC	3' UTR	120	Cell culture strain Indonesia and Guiana
			Deng_1_R	CTA GTC CTT ACC ACC AGG GT			
			Deng_1_P	ACA GCT TCC CCT GGT GTT GGG CC			
			Deng_2_F	AGA AGA GAA GAG GAA GAG GCA	NS5	138	Cell culture strain D2
			Deng_2_R	TGG CCT GAC TTC TTT TAA CGT C			
			Deng_2_P	CTT GGA CGG GGC TCA CAG GTA GC			
			Deng_3_F	GAA GAG ATT CAG GAA GGA GGA	NS5	144	Cell culture strain D3
			Deng_3_R	GGC CTG ACT TCT TCT TTT AAC G			
			Deng_3_P	TCC TTG GAC GGG GCT CAC AGG C			
			Deng_4_F	TGG ATT CAG GAA GGA GAT AGG	C	124	Cell culture strain D4
			Deng_4_R	GTT CGC CAT CTC TTG TTG ACA			
			Deng_4_P	CCG CAT GCT GAA CAT CTT GAA CGG GA			
		Japanese Encephalitis	JEV_F	GCT TAG CGC TCA CAT CCA CT	NS2a/Ns2b	88	Cell culture strain SA14
			JEV_R	CAC CCT CTC TTC TTG TTT GGG			
			JEV_P	TGC AGA CCA TTA GTC CGG CAG CTA TAG T			
			JEV_IIIa_F	TGG ACG TCC GCA TGA TCA AC	E	162	
			JEV_IIIa_R	CCT TGT TTG CAC ACA TAG CTA C			
			JEV_IIIa_P	TAT CAG CTC GCT TCT CGT TGT GGG CTT			
			JEV_IIIb_F	ACT GAC ATC TCG ACG GTG G	E	131	
			JEV_IIIb_R	CAT GTG TCA ATG CTT CCC TTC			
			JEV_IIIb_P	CTC GGT GCC CCA CGA CTG GAG AA			
			JEV_II_F	GAA GGA GCT AGT GGA GCT AC	E	156	
			JEV_II_R	TGA AGC GTG ATA GCA GTA GCT			
			JEV_II_P	TTG GGT GGA CCT GGT GTT AGA AGG AGA TA			
			JEV_I_F	GTT ACT GCT ATC ACG CTT CAG	E	163	
			JEV_I_R	GTC AAT GCT TCC TTT CCC GAA			
			JEV_I_P	TCA CTG ACA TTT CAA CGG TGG CTC GAT G			
			JEV_IV_F	TTG AAA GGT GCC CAA AGA TTG G	E	188	
			JEV_IV_R	TAT TCC CAT CCA GAG TAG CAG A			
			JEV_IV_P	CAG CGT TGG GAG ATA CAG CTT GGG ATT			
			JEV_V_F	GGA TGG ATG CTT GGC AGC AA	prM/E	170	
			JEV_V_R	GAG GCA ACT GTC TCC TTC CA			
			JEV_V_P	CAA CGG CCA GCG TGT GGT GTT CAC			

Ilheus	IlheusV_F	GGA AGT GCC ATT TTG CTC CC	NS5	77	Cell culture strain PE 20545
	IlheusV_R	AGC GGC ATG GAA CTA CGA TG			
	IlheusV_P	TAC GTC CAT CCT TCA TCA ACA GCT CGT TG			
Kedougou	KedV_F	AAG CAC CAA CGG CAG AAG TG	E	85	Cell culture strain Dak Ar
	KedV_R	TGG CTG AAG TCC AAG CCT G			
	KedV_P	ATT GAG CCT CGC ACG TCAT GGT CAT AGT			
Kokobera virus groupe	Kok_I_F	CAT CAA GGT ACC AGT GAA CGT	NS5	144	Cell culture strain MRM
	Kok_I_R	AAC TCC TCG GAA CTC AAC TCT			
	Kok_I_P	CTT CTC ACT CTC ATA CCT CTT CTG TGT TCC			
	Kok_II_F	GTT GTG ATA GTC AGG CCT GAA	3'NCR	86	
	Kok_II_R	CCA CGA CAC TGG AGC ATC A			
	Kok_II_P	CTC CAG GCC GGA TGC AGG CAG			
	Kok_Bai_F	ACA GAA GAG GTA TGA GAG TGA G	NS5/3'NCR	109	
	Kok_Bai_R	TCA GGT GGC TTT TCA GGC CT			
	Kok_Bai_P	AAG AGA GTT GAG TTC CGA GGA GTT CTG TAG			
	Kok_Tor_F	TGC GGA TCT CTC ATC GGC TA	NS5/3'NCR	183	
	Kok_Tor_R	TCA CCA TCA TTC CCT CTT ACA G			
	Kok_Tor_P	AAC CCC TGT GAA CTT GAC ACT TTC CTC C			
	Kok_Map_F	AAG AGA AGA TCT ATG GTG TGG C	NS5	81	
	Kok_Map_R	CTC CAC AGT TCT TCG GAT GTT			
	Kok_Map_P	TCG CTC ATT GGA CAC AGG CCC AGA A			
	Kok_Strat_F	AAG GCA GAA GGC CGC ATC AT	E	177	
	Kok_Strat_R	AGT CTC CCA AGT CTG CCG T			
	Kok_Strat_P	CCA GAA GGA GAA TTT GGA GTA CAC AGT GC			
Koutango	Kout_I_F	GAG ATT CCT CGA GGG AGA G	NS5	200	Cell culture strain DAK AR D
	Kout_I_R	ACA AGT TGC ACG GCC AGG TT			
	Kout_I_P	CAC CGC TTA CTA GCC CGA GCA ATC ATT			
	Kout_II_F	CAG AGC ATC AGG TAA CAT CGT	NS5	190	
	Kout_II_R	TGA GCC TCT CGA TTC TCC TC			
	Kout_II_P	CCA CGC TGT GAG CAT GAC CAG TCA A			
Murray Encephalitis	EnM_gI_F	TTA CCT ACA CTG ATC TAG TGC G	NS1/NS2a	176	Cell culture strain 3329
	EnM_gI_R	CCA GGA CGA GAT TCT CTT GAT T			
	EnM_gI_P	AGT CCA TCT ACT GCG TGT CAA GCT GG			
	EnM_gII_F	ATG TGC GAT GAC ACC ATC ACT T	prM	117	
	EnM_gII_R	TCG TGC ATC TTC CAT AGT TCA C			
	EnM_gII_P	ACG AAT GTC CGA AAT TGG AAA GTG GAA ACG A			
	EnM_gIII_F	ACG ATG AGT CCA CTC TGG TAA	NS1	114	
	EnM_gIII_R	TTC CTC AAG ACC TCC TGG GT			
	EnM_gIII_P	AG TCA AGG GTC CAA GCA TTC AAT GGA GAC A			
	EnM_gIV_F	TTG CGA GCA GTA CAC TCA AAC T	E	95	
	EnM_gIV_R	TGC ACA TCC CAT AAG TAG TTC C			
	EnM_gIV_P	CAC CTC AGG CCA TCT CAA GTG CCG			
	EnM_gV_F	GGA AGA ATG GTG ACG GCT AAT	E	127	

	EnM_gV_R	TGA TTG ATC TGC TTG TCT CCC			
	EnM_gV_P	CTG CCT ACC ACG ATG TAT GAG TCT CC			
Rocio	RocioV_F	GAA AGG AAG CCT GCA GAC CT	E	72	Cell culture strain 5P H34 675
	RocioV_R	TCA CGC TGG ATG GTC ATT CC			
	RocioV_P	ATA GCT TTC TGC GTG CAT CCA AAT TTA ACG C			
Saint Louis Encephalitis	StLouisEV_F	TGCTGATGTTGATTGCCCCG	prM	125	Cell culture strain MSI-7
	StLouisEV-R	GTGACACAGCTTCCTCCTTC			
	StLouisEV-P	AGTACCAAGTCAATCCATGTTGCCCCG			
Spondweni	Spond_I_F	CTG GTT AAG CAG AGA GAA CTC	NS5	156	Cell culture strain SM-6 V-1s
	Spond_I_R	TTC TAG GTC GCA TTT CGT GAT G			
	Spond_I_P	TGG AGG GGG AGT TGA GGG CTT GG			
	Spond_II_F	AGG AGT GTG AAT GCC ACC AG	NS5	190	
	Spond_II_R	TGC CAA GTG GAT CCA TAT TCC T			
	Spond_II_P	CCA GCT GCT CAT GCA CAG AAT GGA CAT			
Usutu	UV_F	CAC GCA ACA TGG GAA AAA CC	prM	96	Cell culture strain SAAR-1776
	UV_R	GCA TCC AGT TTG GGG CAT TC			
	UV_P	TGC TGG ATT AGA GCC ATG GAT GTC GGG TA			
Wesselsbron	WessV-F	GTG TCT CCT GGA AAT GGA TGG	NS5	79	Cell culture strain SAH-177
	WessV-R	CAT CAA CAG CCA CAT TTG CG			
	WessV_P	ATG ATC AGA GAA ACG GCC TGC CTC AGT			
West Nile	WN_F	AAG TTG AGT AGA CGG TGC TGC	3'NTR	92	Cell culture strains UG956 D117 + IS98 + MRM16
	WN_R	AGA CGG TTC TGA GGG CTT AC			
	WN_P	CGA CTC AAC CCC AGG AGG ACT GG			
	WN_1A_F	GTT GGC TCT CTT GGC GTT CT	C	194	Cell culture strain IS98
	WN_1A_R	GCA ATT CCG GTC TTT CCT CC			
	WN_1A_P	TCA GGT TCA CAG CAA TTG CTC CGA CC			
	WN_1B_F	GAA GTT AGC AGT CTA CGT TAG G	prM	194	Cell culture strain Kunjin MRM16
	WN_1B_P	TAT GGA AGA TGC ACC AAG ACA CGA CAC TC			
	WN_1B_R	GCA TAT CCA GGG TTT CTC AAG			
	WN_1C_F	TCA TGG TTG CGA CGT TCG TG	NS2a	188	NA
	WN_1C_P	AAG GCT AGG TGG ACG AAC CAG GAG AA			
	WN_1C_R	AAG TGT TGG TAA ACG TGA TGG C			
	WN_3_F	ATT TGA AGA ACC ACA TGC CAC G	E	195	Cell culture strain Rabensburg
	WN_3_P	AAG CAA TCG GTG GTC GCC TTA GGT TCT			
	WN_3_R	TGC GCA TAC TCC ATA GGT CG			
	WN_4_F	GAT TGT GAA CCC AGG TCA GG	E	137	NA
	WN_4_P	CGT TGA TGT GGA CGC CTT CTA CGT GAT			
	WN_4_R	TGT TCC TCC AGT TCG TGT TTC			
	WN_2.1_F	GAG CTG TTT CTT AGC ACG AAG	C	137	Cell culture strain UG956 D117
	WN_2.1_P	ATC TCG ATG TCT AAG AAA CCA GGA GGG C			
	WN_2.1_R	CAG ACT CAG CAT AGC CCT CT			
	WN_2.2_F	CAT GGA GAA AGT ACA CTG GCT A	prM	183	
	WN_2.2_P	ATA AGA AAG GAG CTT GGC TGG ACA GCA C			

			WN_2.2_R	GCA GTA GGA TAG CGA ACA CG			
		Yellow fever	YF_F	CTG TCC CAA TCT CAG TCC AAG	prM	69	Cell culture strain 17D
			YF_R	AAC GTT TTC CAC CCC ATA GC			
			YF_P	AGA GGA GCC AGA TGA CAT TGA TTG CTG GT			
			FJ_AO_4_F	TGA TGA AGT GCT GAT TGA GGT G	E	142	
			FJ_AO_4_R	TCC GCG CCT TTC ATG GTC T			
			FJ_AO_4_P	AAC CCA CCC TTT GGA GAT AGC TAC ATC AT			
			FJ_AO_3_6_F	AGA CCC GGC AAG AAA AAT GGA	M	169	
			FJ_AO_3_6_R	AGC CAA GAC CAG TAG GGC AA			
			FJ_AO_3_6_P	TGA CTG GAA GAA TGG GTG AAA GGC AAC TC			
			FJ_AO_1_F	CCA TGA GCT TGT TTG AGG TTG A	E	185	
			FJ_AO_1_R	GCA TTC CAG AGT AGC TTT TCC A			
			FJ_AO_1_P	CCA GAC AAA GAT CCA GTA CGT CAT CAG AG			
			FJ_AC_AE_F	TGG TCG AAA AGC TCA GGG TAA	C	116	
			FJ_AC_AE_R	GAA GGG CCA GGT CTG TTT C			
			FJ_AC_AE_P	AAC CCT GGG CGT CAA TAT GGT AAG ACG			
			FJ_AmS_2_F	CAC ATT CCA GGA TAC AAG GTC	NS1	158	
			FJ_AmS_2_R	AAT GAT CTT CCC ACT GTC GGT			
			FJ_AmS_2_P	CAG ACA AAT GGG CCT TGG ATG CAG GT			
			FJ_AmS_1_F	AGC TGA GAT GGG AGC CAA TC	NS3	170	
			FJ_AmS_1_R	TCT CTG TTA GGG TTC CTT CCA A			
			FJ_AmS_1_P	TCT GCG TGG AGA GAG TGT TGG ATT GTA G			
		Zika	Zika2_F	AAT GAC ACA TGG AGG CTG AAG	NS1	70	Cell culture strain MR766
			Zika2_R	TGT GTG AGA CTT TGG CCA TTC			
			Zika2_P	AGG GCC CAC CTG ATT GAG ATG AAA ACA TGT			
			Zika_III_F	TGG CAG TGC TGG TAG CTA TG	NS1/NS2a	78	
			Zika_III_R	GTG GCA CCC ATC AAA ATT GCA			
			Zika_III_P	AGC TTA GCC AGG TCA CTC ATT GAA AAT CCT			
			Zika_IV_F	CCA ACT GGG AGA ACC ACC T	NS5	102	
			Zika_IV_R	GGT CGT TCT CCT CAA TCC AC			
			Zika_IV_P	ACT CTA TTC CAC ACC ATG AGC ATG TCC TC			
			Zika_VI_F	GCA TCA GGT GCA TAG GAG TC	prM/E	194	
			Zika_VI_R	TTG ATG CCT CAT AGC AGT AGG A			
			Zika_VI_P	AGC AAT AGG GAC TTT GTG GAA GGT ATG TCA			
			Zika_VII_F	GCA ATC AAG CCA TCA CTG GG	C	138	
			Zika_VII_R	GTC TCT TCT TCT CCT TCC TAG			
			Zika_VII_P	CAT TGA TTA TTC TCA GCA TGG CAG CCA GAT C			
			Zika_VIII_F	AAG ATC CTA CTG CTA TGA GGC A	E	198	
			Zika_VIII_F	GGA GCA TGC AAA CTT AGC GC			
			Zika_VIII_F	TCA ATA TCA GAC ATG GCT TCG GAC AGC C			
Phenuiviridae	Phlebovirus	Rift Valley fever	RVFV_F	ACAAAAAGCGGGTGGGGATAG	Nsm/Gn	82	Cell culture strain ZH548
			RVFV_R	GCAATCCCTGCCATGGTTTC			
			RVFV_P	CGGTGTGAGAGACGAAGAGACGCTAAG			

			RVF_SegS_F	GTT GAT TTG CAG AGT GGT CGT			
			RVF_SegS_R	GCG AAC CTC GTG ACT AGG A	NSs	134	
			RVF_SegS_P	CGA TGG TGC ATG AGA AAG ACA CAA CAG G			
Peribunyaviridae	Orthobunyavirus	Batai	batai_1_F	GAA TGG GAG GTT ACG CTT AAC			NA
			batai_1_R	GTA CCT GGC AAG GAA TCC AC	N	134	
			batai_1_P	CTT GGG GGC TGG AAG GTT ACT GTA TTT AAT A			
			batai_2_F	GGG CAG ATG GTG AGG AGA T			
			batai_2_R	TGC TAA CCG TCC ATG TCC CT	N	188	
			batai_2_P	TTA CCT CTC ATT CTT CCC AGG CTC GGA			
		Bunyamwera	Bunyam_1_F	CGG TAC CTA CTT GAG AAG ATT C			NA
			Bunyam_1_R	TAC ACC TCT TCT CCA TCT GAC	N	121	
			Bunyam_1_P	TGA AAG TGA GTG AAC CAG AAA AGC TGA TCA TC			
			Bunyam_2_F	TTG TTA CAG CCG GTG GTA GTA			
			Bunyam_2_R	GAC TTA AAC CAG CCA GTG CTA A	Gn	192	
			Bunyam_2_P	TGG TGG TTC CTG TCA CTT CAA AAT GGT TCA			
			Bunyam_3_F	ACA TCA CTC TTC GGT GCA GG			
			Bunyam_3_R	TGG ATC CTC TAG CAG CCC A	NSm	200	
			Bunyam_3_P	TCT GAC CAT CAT TTT TGC AGG AGT AGC ATT G			
		Bwamba	Bwa_S_F	TAT CCA GGG GCA CTC AAT ACA A			Cell culture strain BWA
			Bwa_S_R	TCC ACC TCC CAG TTT CCA AAT T	N	124	
			Bwa_S_P	ATA CCG CTA GGA CAT TCT TCC TCA ATG CC			
		Cache Valley	CacheV_S_F	GAT GGT CTT ACC CTC CAC AG			NA
			CacheV_S_R	TAA GAA CAT CTC TGA GCC AGG	N	194	
			CacheV_S_P	ACT CAG TGG ATA CCT TGC CAG GTA CCT A			
		California Encephalitis (Snowshare, Chatanga, La Crosse viruses)	CalifEV_1_F	GCA TCA ACA GGT GCA AAT GGA T			NA
			CalifEV_1_R	TCG CCA AAT TTA GGA CTT GCC	N	172	
			CalifEV_1_P	TTG ATC CTG ATG AAG GGT ATA TGG CAT TCT G			
			CalifEV_2_F	GGC AGA GGT ATG GTT CAC TAA			
			CalifEV_2_R	GTC CTA AAC AAT TTG CCT GCC	N	186	
			CalifEV_2_P	CTG CTG AAA AGT GGA TGT CCC AAA AGA CC			
		C Groupe_Apeu	GpC_Ape_F	CCC AAA CTA CCA AGA GTG TAA C			NA
			GpC_Ape_R	GCA AGC ATA CGT CTT TGG GAT A	G2	102	
			GpC_Ape_P	AGC ATA AGG TCT CGG TCG GGC TTG AAA			
		C Groupe_Restan	GpC_Rest_F	GTA AGA GCA GAG GAT CAG CAT T			
			GpC_Rest_R	TAT ACT CCT GAC ATT GCC CAG A	G2	175	
			GpC_Rest_P	TTG CCT TGC AGT AAT TCT AGC CAC ATT ACT C			
		C Groupe_Murutucu	GpC_Muru_F	CTA GGA TTC TCT GCA AGA GTA G			
			GpC_Muru_R	TGT TGT ATG ACA GCT GCA TTG C	G3	110	
			GpC_Muru_P	AGG TTC TGC ATT TTG CCT CGC TGT GAT ATT A			
		C Groupe_Oriboca	GpC_Oribo_F	CTA GAA CTG TAT GCT GAC ACA G			
			GpC_Oribo_R	TCT CTC CCT TTT GAG CTC TGT A	N	198	
			GpC_Oribo_P	AAC ACC GCC CAG AGA TTG AAG AGA AAA TCA			
		C Groupe_Marituba and Apeu	GpC_Mari_F	GTT CTT CCT CCG TGC GAA TG	NSs	140	

	GpC_Mari_R	CCA TCA GCG ACC GTA TTT GC			
	GpC_Mari_P	AGG CTA AAC AGA AGC TCC GTA AGA GTT CG			
C Groupe_Nepuyo	GpC_Nepu_F	GAG TGC ATC TAC TTT TGA CCC TA	NSs	147	
	GpC_Nepu_R	AGC GAC CTT TGC CGT ACT C			
	GpC_Nepu_P	AA CAG GCG TAC CAG AGT TTT ATC GAT AAC C			
C Groupe_Itaqui	GpC_Itaq_F	AT TGG TCT GAG TGT AAC CCT G	N / NSs	194	
	GpC_Itaq_R	CTG TTG TCG TAC CCA TAA CCT			
	GpC_Itaq_P	TGG AGA CAG CCA ACG GCC CGA TC			
C Groupe_Caraparu	GpC_Cara_I_F	TTC TGC CGT CGT ACG AGT CT	N / NSs	189	
	GpC_Cara_I_R	GTA ACC TGA TAT GCG ATG CAA C			
	GpC_Cara_I_P	TCT ACA TCA ACG CGG CGA AGG TCA AAG			
	GpC_Cara_II_F	TGA GAG TGA CTA CGG TTC CC	N / NSs	187	
	GpC_Cara_II_R	CGT AGT CAG GCA GTG GAA TG			
	GpC_Cara_II_P	AAT TGG AGT CCG CTA TTG TTC GAG TCT TCT A			
Germiston	Germi_segS_F	GGG CCA GAA GTC TAC CTG T	N	196	NA
	Germi_segS_R	TCC ACT TCT CCA AGC TTA GTT G			
	Germi_segS_P	CCT TCT TCC CAG GTG CTG AAA TGT TCC T			
Guama Group_Catu	Guama_I_F	TCA GAG AGG ATG AAG AAG CAC	G2	105	NA
	Guama_I_R	ACA GGA TGA AAG AAG AGC CTC			
	Guama_I_P	AGA GAG TCT GGA ATG TGC CAC GGA TAT AAA T			
Guama Group_Catu and Guama	Guama_II_F	GTC GCA CAA GGG AGA AAT AAC	N	154	
	Guama_II_R	GCT GTA CCA AAC TTA AGA GTG G			
	Guama_II_P	TTC ATC CCT AGT GAG GCT TAC GCT GTC			
Guama Group_Bimiti	Guama_III_F	ACT TCA GAA TGC TCC CAT TGG	N	173	
	Guama_III_R	GGA GCT TAG ACA CTA GTG TCA			
	Guama_III_P	CAA TTG GGA TTT ACA GAG TAC AGC AGA AAC AG			
Guama Group_Moju	Guama_IV_F	AGT TCA GAA TGC TTC CCC TAG	N	102	
	Guama_IV_R	ATA TCG CCA TAC TGC TGT CTG A			
	Guama_IV_P	CAA TAG GCA TTT ACA GAG TCC AGC AGA AAC A			
Guama Group_Mahogany Hammock	Guama_V_F	GGA TCA CCT GGG AAC ATG GT	N	196	
	Guama_V_R	CAG GAC CAT CCA TTG GCT TG			
	Guama_V_P	CTG GCA TGT CTC CAT ACT GCT GGC G			
Guaroa	Guaroa_S1_F	GTT TAA CCC GGA GCT CCA ATA	N	217	NA
	Guaroa_S1_R	AAT CAT CGA GGA CTG GAC TGT T			
	Guaroa_S1_P	TGC TAC ATT TAA ACG TAC AAA CAC AAC AGG GC			
	Guaroa_S2_F	TAT AGC TGC ATC AAA CGG GAT C	N	146	
	Guaroa_S2_R	GCC TCC ATC ATC TTT TTC TGG			
	Guaroa_S2_P	ACA TGG GAA GAT GGA CCA GAG GTT TAT CT			
	Guaroa_S3_F	GGA GAC TTT CAA ATT CTA CCC C	N	185	
	Guaroa_S3_R	ATT AGC TTT CTT CCA GCC CAG			
	Guaroa_S3_P	TGA TGA GAC AAC AGA AAG AGC AGC ATT GAT C			
Ilesha	Ile_L_F	GCA CAT GGC GAT TTT ACA CTG A	RdRp	108	Cell culture strain ILEP1
	Ile_L_R	TCT TGG TGG CAT AGA ACT GAT G			

	Ile_L_P	CTG CAC CAT GGT GCA CAA CAG AGA CT			
Inkoo	Inkoo_S_F	TTG ACG TAG ACC TAC TGA TGG	RdRp	149	NA
	Inkoo_S_R	GTG GGA TCT CTA ATG TCA ATG G			
	Inkoo_S_P	CAA GAC ATG ACT ACT TCG GGA GAG AGT TG			
Jamestown Canyon	Jamestown_F	AAG CCA AAG CTG CTC TCG CT	N	166	NA
	Jamestown_R	AAC CCA TCT GGC TAG ATA TCC			
	Jamestown_P	CGT AAA CCG GAG CGG AAA GCT ACT C			
Keystone	Key_segS_F	AGG GTA TGT GGC ATT TAT GGC	N	120	NA
	Key_segS_R	CCA CTC TCC AAA CTT AGG TGT A			
	Key_segS_P	TAA CCA TGG GGA GTC GAT CAG TCT GTC			
La Crosse	LaCrosse_1_F	GCT GCA AGC CCA GTG TAT CA	Gn	196	NA
	LaCrosse_1_R	TGC CAA TCA GAG ACT AGC CAT			
	LaCrosse_1_P	AAG GTG TTT CCA AGA TGG GGC TAT AGT GAA G			
	LaCrosse_2_F	TTC CAA GAT GGG GCT ATA GTG	Gn	175	
	LaCrosse_2_R	AGT CGT GCC AAT CAG AGA CC			
	LaCrosse_2_P	AAG CAA AAC CCA TCC AAA GAG GCA GTC AC			
	LaCrosse_3_F	AAT CAG AGG TGC CTG CAT TAG	Gc	185	
	LaCrosse_3_R	GGC CAG TAC ACA ATT CAT CAT G			
	LaCrosse_3_P	CTG GGA CAT CTA TCG GGT TCA AAA TCA ATT C			
	LaCrosse_4_F	CCA TTC ACA GAG TGT GGC AC	Gn	134	
	LaCrosse_4_R	CGA CTT GCA CAT GAC TCT GG			
	LaCrosse_4_P	ACA TTG TGT CTG TGG TGC TCG CTA TGA TAC			
	LaCrosse_5_F	TGT CTG TGG TGC TCG CTA TG	Gn/Nsm	104	
	LaCrosse_5_R	TTG CAC ATG ACT CTG GCA GC			
	LaCrosse_5_P	ATA CTT CCG ATA GAA TGA AAC TGC ACA GAG C			
	LaCrosse_6_F	GCC CAG TGT ATC AAA GGT GTT T	Gn	97	
	LaCrosse_6_R	GCT AAC ATC ATC TTT CAG GCA C			
	LaCrosse_6_P	CCA AGA TGG GGC TAT AGT GAA GCA AAA CC			
	LaCrosse_7_F	CTC ACA TTT GCA AGA GAG AGG	Gc	123	
	LaCrosse_7_R	CAC TTC TTC GGT CTC CTT CC			
	LaCrosse_7_P	ACA AGT TCA TGG GGA TGC GAA GAG TTT GG			
	LaCrosse_8_F	ATC CAA AGA GGC AGT CAC GG	Gn	199	
	LaCrosse_8_R	GAT CAT CAC CAA CCT CTA TCA C			
	LaCrosse_8_P	AGG TGT GCC TAA AGG ATG ATG TCA GTA TGA T			
Ngari	Ngari_segS_F	TTC ATG ATG TCG CTG CTA ACA C	N	79	NA
	Ngari_segS_R	CCC AAG GTT AAG TGT AAC TTC C			
	Ngari_segS_P	CAG CAG TAC TTT TGA CCC AGA GGT CG			
Nyando	Nyando_1_F	CGC TCG GAT CTT CTT CCTCA	N	154	NA
	Nyando_1_R	TGT AAG ATC CGT GTC GCT GAT A			
	Nyando_1_P	ATG CCC GGA AAG CCA AAG ATC AAC TCT CT			
	Nyando_2_F	CAG AGC CGA AGG TTG GTC TT	N	182	
	Nyando_2_R	TGC CAG CTT GTG CAA CAC TAT T			
	Nyando_3_P	AAA TTT GGA ACA TGG CAG GTG GAA GTG GTC			

		Oropouche	Oropou_F	GTG GTA ACC TCT TCA AGG AGA	Gn	181	NA
			Oropou_R	TAG CAC TGG ATT GCA CTC AGA			
			Oropou_P	TGA ACT TGA GTG TAG GAC TTG GCG AAA TAT G			
		Snowshoe hare	Snow_segS_F	GAC GAT CTT ACC ATC CAC AGA	N	151	NA
			Snow_segS_R	CCG CTA TCC CAT CTC ACT C			
			Snow_segS_P	TTG TCA GGA TAT TTA GCC AGA TGG GTT CTT G			
		Tacaiuma	Tacaiuma_F	CCA TTA GCT GAG GTT GCT GG	N	139	NA
			Tacaiuma_R	TCC TTT TTC ACC CTA GCT ATG C			
			Tacaiuma_P	TGT CTC ATG GGC TAA TTC TAC GCC AGA AAT			
		Tahyna	Tahy_S_F	ATT TGG CTA GAT GGG TGC TAG	N	156	Cell culture strain 92
			Tahy_S_R	GTC CCA CCT GAT CCC ATT AG			
			Tahy_S_P	ACT CAG CTA TGG GGT TGA TAA CGG TTG TT			
		Trivattatus	Trivatta_F	CCA TCA ACA GGT GCA AAC GG	N	199	NA
			Trivatta_R	TGA TTA TTG ACC ACC TCC ACC			
			Trivatta_P	ATT TGA TCC CGA TGC AGG GTA TGT GGC			
Reoviridae	Orbivirus	Orungo	Orungo_1_F	GTG ATT CGG GCA AAC TGC GT	RdRp	171	NA
			Orungo_1_R	GAA CTC GCA TAA TTG CGC AGA A			
			Orungo_1_P	TTT AAA GGT ATG CAA GTT GTG GTA GAG TCG AC			
	Seadornavirus	Banna	BannaV_F	ATCCGGTGTCGTCACTTGG	VP7	110	Cell culture strain BAV
			BannaV_R	TCAAAGCCCACACACTCAGTG			
			BannaV_P	ACCGTGACTACACCATGCCACTACCAAATG			
Rhabdoviridae	Vesiculovirus	Jurona	Jurona_F	AGT TGA TGA TTA CAG AGG ACC C	N	189	NA
			Jurona_R	TTC TGC CTG ATT GCT TCT AAG C			
			Jurona_P	ATA CCG GAT GGC AAG TCA AGC AGT GG			
Togaviridae	Alphavirus	Barmah Forest	Barmah_F	GTG CCC AGG TCC GAA GTT A	E2	110	NA
			Barmah_R	TCT GTA TGT CAC TTG CGG TTC A			
			Barmah_P	CGG AGG TGA AAG GAA AGA TCC ATG TGC			
		Chikungunya	chik_F_SG	TGG AAT GGC TGG TTA ACA AGA TAA	NSP2	114	Cell culture strain LR2006_OPY
			chik_R_SG	CTC CGC GGA CAC CTA ACG			
			chik-P2	ACG GCC ACC ACG TGC TCC TGG T			
			Chik_WAfri_F	CGA ACT ACA TAT CCG GCA CC	NSP4	129	
			Chik_WAfri_R	TGA TTT GGT ACG ACG CAA CTG T			
			Chik_WAfri_P	AGT GTA CTC ACC CCC AAT CAA TAT CCG AC			
			Chik_Asia_F	TGA TCA AAT GAC CGG CAT CCT	NSP1	104	
			Chik_Asia_R	CGT TGC GTT CTG CCG TTA AC			
			Chik_Asia_P	TGC TAC AGA AGT CAC GCC GGA GGA TG			
			Chik_IndECSA_F	AGG AAG TCC ACG AGG AGA AG	NSP3/NSP4	158	
			Chik_IndECSA_R	TTA GTC TCT GGA TGA TTG CTG C			
			Chik_IndECSA_P	TGT TAC CCA CCT AAG CTG GAT GAA GCA AAG			
			Chik_ECSA_F	ACA CAA CCC CGT TCA TGT ACA	NSP1	191	
			Chik_ECSA_R	CTA CTG AGA ACA GCA CAC GG			
			Chik_ECSA_P	ATG CCA TGG CGG GTG CCT ACC C			
		Eastern Equine Encephalitis	EEEV_F	GAA GTG GCC TGG AGC TTT TG	NSP3	83	Cell culture strain H178/99

	EEEV_R	GCA TGG ATG ACG TTC GGA GA			
	EEEV_P	ATG CTT GAC GAG GTG CGC TTT ACC AGT			
	EEEV_1_F	TGT ATT GCC TCT AAG GCC GC	NSP1	184	
	EEEV_1_R	GTT CGT ACA CCT TTC AGC GC			
	EEEV_1_P	CTG GTA GTA AAT GGA AGT CGG TGC ATG CA			
	EEEV_2_F	GTT CAT GTT GAC TTA GAC GCA G	NSP1	149	
	EEEV_2_R	ACC TGG TCT GTA TCC ACT TCT			
	EEEV_2_P	ACA GCC CAT TCG TCA AGT CAC TGC AAA G			
Mayaro	Maya_I_F	ATG AGC GAA GCC TAC GTG G	E1	162	Cell culture strain TC625
	Maya_I_R	ATC GTT ACG GCA TGG TCA CC			
	Maya_I_P	AGC GCG CTG ACG TGT GTA AAC ACG A			
	Maya_II_F	CAG CCG GCA ATA TCC ACG T	E1	194	
	Maya_II_R	GTC TAG TGA ATG CGC TGT CG			
	Maya_II_P	CCC CTA TAC CCA GAC ACC ATC TGG TT			
O'Nyong-Nyong	ONN-9237F	ACA AGT GCA AAT GTG ACG GC	E2	125	Cell culture strain Dakar 234
	ONN-9362R	GCG GTG AAT TGT ATT GCC ATT T			
	ONN-9307P	GTT TGT AAC CGC TGT GTG GCA TTG GTC T			
Ross River	RRV - 9,239 F	CAA TGC CAT GCT GCC GTT AC	E2	72	Cell culture strain 5281v
	RRV - 9,328 R	TTT GCC CCT CCT AGC TGT C			
	RRV - 9307 P	ATC AGC CCT GGG AAC AAA TGG AGA GGT			
Semliki forest	SemFV-9240F	CGT CAT CCA CGG CAA AAG AG	E2	72	Cell culture strain 1745
	SemFV-9310R	TGC GGT AGG AAA AGA GCG T			
	SemFV-9261P	TGACACTGCACCTTCACCCAGATCATC			
	Seml_II_F	AAC AGC TGA AGA CAA GCA GGA	NSP4	179	
	Seml_II_R	AAC GCC TTA ATG TCC CTC GC			
	Seml_II_P	CGA AGA CAG GCG ACG AGC ACT GAG			
Sindbis	SinFP	GGT TCC TAC CAC AGC GAC G	NSP1	75	Cell culture strain Egypt 339
	SinRP	TGA TAC TGG TGC TCG GAA AAC			
	SinP	TTG GAC ATA GGC AGC GCA CCG GCT			
	Sindbis_I_F	GAA GGT AGA CGC CTA CGA AC	E1	152	
	Sindbis_I_R	ATG TAC TCT TGG TTG GTG GAA G			
	Sindbis_I_P	ATG CGA CCA CTG TTC CAA ATG TGC CAC			
	Sindbis_2_3_F	GGT AGA CGC CTT CGA ACA TG	6k	152	
	Sindbis_2_3_R	GTG ACG TAC TCC AGG TTC GT			
	Sindbis_2_3_P	CGA CCA CTG TCC CAA ATG TGC CGA G			
	Sindbis_IV_F	AAT TCG AGG TAG TAG CAC AGC	NSP1	176	
	Sindbis_IV_R	GCA GAC GCA GTG ATA GTG GT			
	Sindbis_IV_P	AGG CCA CAC CAA ATG ACC ATG CTA ATG C			
	Sindbis_V_F	GTA GGA ATT AGG AAC ACT CTC G	NSP4	162	
	Sindbis_V_R	CTG ACT ATT TAG GAC CGC CG			
	Sindbis_V_P	CAG TTG CCG TAT CGA CCA GGT ACG A			
	Sindbis_VI_F	TAA CAG TGA AGA CGT GGT CAC	NSP2	198	
	Sindbis_VI_R	CTC CGT CTC TTG TTC CTT CAT A			

	Sindbis_VI_P	CGC TCT GGC CAG AAA GTT CGT CCG			
Una	Una_1_F	ACG TGC ACA GCA TCG TGC A	E1	155	NA
	Una_1_R	TAG TGC AAC TGC TGT CAG CG			
	Una_1_P	CGC CAC CAA AGG ACC ACA TCG TAC C			
	Una_2_F	CCG CAG TAA GTG CCT TAC AC	E1	187	
	Una_2_R	TGA TTA ATA GCA CTG CGA CCG			
	Una_2_P	CAT ATG CAC TCA CAC CAG GCG CAG T			
	Una_3_F	CGT ATG CTC TCA CAC CAG G	E1	184	
	Una_3_R	ACG GAG GCA GTA CGA AAT GAT			
	Una_3_P	TGC GGT GAT TCC CAT GAC AGT TGG ACT			
	Una_4_F	CAC GAT CAT GCG GCT GCTT	E1	139	
	Una_4_R	ATG AAT CGG GTC CCT GCG A			
	Una_4_P	ATA AGG CAC ATA CCG CTT CAA TGA AGG CC			
Venezuelan Equine Encephalitis	VEEV -5306 F	CATCCTTGACACCCTGGAGG	NSP3	89	Cell culture strain TC83 vaccine
	VEEV -5394 R	GAAACTCCATGCTCCTTGCG			
	VEEV - 5345 P	AAGTAAGAGTTAGTCTCGGCTGACGCTG			
	VEEV_1_F	GCT ACG ACC GCA AAC CAA C	E3/E2	192	
	VEEV_1_R	CAG CTT CCT ACA GCA CAC CT			
	VEEV_1_P	TGA AAC CTT GGC TAT GCT CAG CGC CA			
	VEEV_2_F	GTT ATG ACG AGT TGC TCG AAG	E3/E2	176	
	VEEV_2_R	CAT GCC CTT CGC TCC TTA C			
	VEEV_2_P	CAG TAC TGA AAT GTC CAG GCA GAG GCA A			
	VEEV_3_F	GCT GCC ATA GTC CAA TAG CAA	E2	186	
	VEEV_3_R	CGA GAT GTG TGG AGT GAC AC			
	VEEV_3_P	T TGA GGC AGT GAA GAG CGA CGG GC			
	VEEV_4_F	ACG GAG TAG AGC AAG CGT G	E2	172	
	VEEV_4_R	ACA CTC GCA TTC CAC CAG G			
	VEEV_4_P	CCA AGT CTA CGC ACA TGA TGC ACA GAA C			
	VEEV_5_F	TTT GCT ATG ATC GGA AAC CAG C	C/E3	164	
	VEEV_5_R	AAC TCC CAA CGG CAC ACC T			
	VEEV_5_P	AGA GAC GCT GGC CAT GCT CAG TGC			
	VEEV_6_F	CCC TTA CAT GGC CAG ATG CA	E3/E2	199	
	VEEV_6_R	GTA GCG GTA TCT CTT CAA TGG T			
	VEEV_6_P	TCA GAT GTG CCG TTG GGA GCT GCC			
	VEEV_7_F	AAA CTT ACA CGT CCG TAC ATG G	E3	173	
	VEEV_7_R	CCT CAT AAC TCT GCT CTT AAC G			
	VEEV_7_P	CCA AGT GTG TGC GGT GTG CCG TTG			
	VEEV_8_F	TTG AAG CCG TAT TGA AGT GTC C	E3/E2	100	
	VEEV_8_R	TCG GAC GCA CTT TGC CAT GT			
	VEEV_8_P	AGG TAG GCA GAA GAG ATC CAC GGA AG			
	VEEV_9_F	GGA CTC ATC CGG AAA CTT GAA	E3/E2	147	
	VEEV_9_R	GCA ACG AGC TAG GAG GAA GT			
	VEEV_9_P	AGG AAG AAC AAT GAG GTA CGA CGT GCA AG			

	VEEV_10_F	GCT GTT AGA TGG AGT ACT GAG A	E3/E2	182	
	VEEV_10_R	AAG CGC ACG TAC CCA TCA TG			
	VEEV_10_P	TGT CAA GGG AGG TCC AAG AGG TCC C			
	VEEV_11_F	ATA TAA GCT TAC CAC GCC GTA C	E2	169	
	VEEV_11_R	ATT GCC TGA TGA ATC TAG TCC G			
	VEEV_11_P	ATG GCC AGG TGC TCC AGA TGT GCA G			
	VEEV_12_F	GAT CCA CTG ATG AGC TGT TCA	E3/E2	185	
	VEEV_12_R	TGA CGC TTC CCG ATG GAT C			
	VEEV_12_P	AAG AGT ACA AGC TCA CAC GGC CAT ATA TG			
	VEEV_13_F	GGG ACT CAT TGA CCA TGG AG	E2	190	
	VEEV_13_R	AGG GAG GTG CAT TTC GAC GT			
	VEEV_13_P	TTC AAG AAA GAT ACA GTA ACG CAC TCA TGC TC			
Western Equine Encephalitis	WEEV- 4,164 F	GGACGGCTAGACTTGTGAAG	NSP3	115	Cell culture strain 47a
	WEEV - 4,278 R	ATGCTCATGTAGGCAGCTGC			
	WEEV - 4,184 P	CACGAACCGCTCATCATACATGCTGTA			
	WEEV_B2_B3_F	GGA GCG TAT ATT TTC TCA TCG G	NSP3	111	
	WEEV_B2_B3_R	GGC GTA ATA CTT CTC ATG GAC			
	WEEV_B2_B3_P	AAA CAG GCC AAG GTC ACC TTC AAC AGA AAT			
	WEEV_B1_A_F	TGT TCA AGA CTT TCA GGC ACT G	NSP2	159	
	WEEV_B1_A_R	TTC TGA GTC TGT GTC CTG AGT			
	WEEV_B1_A_P	AGT GAG AGC GCC ACG ATC GTT TTC AAC			
Beta-actine	beta-actine_F	GCT ACG TCG CCC TGG ACT T	beta-actine	151	Mosquitoes
	beta-actine_R	AGG AAC GAC GGC TGG AAG A			
	beta-actine_P	AGG AAA TGG CCA CCG CTG CCT CGT			
Escherichia coli	eae_F2	CATTGATCAGGATTTTTCTGGTGATA	eae	102	Culture of EDL933 strain
	eae_R	CTCATGCGGAAATAGCCGTTA			
	eae_P	ATAGTCTCGCCAGTATTCGCCACCAATACC			

NA: not available.

Table S2. Primers used for full genome sequencing of Zika virus.

Name	Sequence (5'-3')	Sense Antisense	Primer concentrations
ZIKV- 1Sbis3	AGTTGTTGATCTGTGTGAGTCAG	Sense 1	10 µM
ZIKV-epidemic-947R	AATCAGCAGTATCATGACCAAGT	Antisense 1	10 µM
ZIKV-epidemic-889S	TTAGCAGCAGCTGCCATCGC	Sense 2	10 µM
ZIKV-epidemic-1893R	GGTACACARGGAGTATGACACG	Antisense 2	20 µM
ZIKV-epidemic-1795S	GCTGGAGCTCTGGAGGCTG	Sense 3	10 µM

ZIKV-epidemic-2753R	ATCCCACAACGACCGTCAGTT	Antisense 3	10 µM
ZIKV-epidemic-2718S	GGAGCTCAACGCAATCCTGGA	Sense 4	10 µM
ZIKV- 3844Rbis	TGTCCAATTAGCTCTGAAGATG	Antisense 4	10 µM
ZIKA_3581S_cam	AGTGCTTGTGATTCTGCTCATGGT	Sense 5	10 µM
ZIKV-epidemic-4581R	GTACCACGCTCCAGCTGCA	Antisense 5	10 µM
ZIKV-epidemic-4535S	TGGTCCTGATGACCATCTGTG	Sense 6	10 µM
ZIKV-epidemic-5610R	GGTGTCCATAATTGGTGAGTTG	Antisense 6	10 µM
ZIKV-epidemic-5432S	TACTACAGCCAATYAGAGTCC	Sense 7	20 µM
ZIKV-epidemic-6545R	CGAGGTTGTCAATGGCTTCCT	Antisense 7	10 µM
ZIKV-epidemic-6404S	CGAGGTGGATGGAYGCCAGAG	Sense 8	20 µM
ZIKA_7511R_cam	CACAAAGTGGAAGTTGCSGCTGT	Antisense 8	20 µM
GP-ZIKV-Cuba-09/2018_6760S	GAGCCAGCCAGAATTGCATG	Sense 8	10 µM
GP-ZIKV-Cuba-09/2018_7893R	CTGCCACATCCAAGATCAATG	Antisense 8	10 µM
GP-ZIKV-Cuba-09/2018_7768S	CTCAAGGACGGTGTGGCAAC	Sense 9	10 µM
GP-ZIKV-Cuba-09/2018_8871R	ATGCTGCATTGCTACGAACC	Antisense 9	10 µM
GP-ZIKV-Cuba-09/2018_8771S	CACTCGTCAGGTTATGAGCATG	Sense 10	10 µM
GP-ZIKV-Cuba-09/2018_10019R	CAGTTGGAACCCAGTCAACTG	Antisense 10	10 µM
ZIKV-7328S	ACGGCAGCTGGCATCATGAAG	Sense 9	10 µM
ZIKV-epidemic-8243R	TGCTGGTGTATGGGCACAACA	Antisense 9	10 µM
ZIKV-epidemic-8166S	AGAAGCACGGACGCTCAGAG	Sense 10	10 µM
ZIKV-epidemic-9171R	CATCCAGTGATCCTCGTTCAAG	Antisense 10	10 µM
ZIKV-epidemic-8963S	CAGTGGAAGCTGTGAACGATC	Antisense 11	10 µM
ZIKV-epidemic-10338R	GTGGATAGGTARTCCATGTAC	Antisense 11	20 µM
ZIKV-epidemic-10248S	TCTCATAGGGCACAGACCGC	Sense 12	10 µM

ZIKV-epidemic-10670R	TCCCTCTTCTGGAGATCCAC	Antisense 12	10 µM
